# Therapeutic effect of *Baccharis anomala* DC. extracts on activated hepatic stellate cells

**Published:** 2019-02-18

**Authors:** Bruno de Souza Basso, Fernanda Cristina de Mesquita, Henrique Bregolin Dias, Gabriele Catyana Krause, Matheus Scherer, Eliane Romanato Santarém, Jarbas Rodrigues de Oliveira

**Affiliations:** 1 PUCRS, Escola de Ciências, Laboratório de Pesquisa em Biofísica Celular e Inflamação, Porto Alegre, Brazil; 2 Graduate Program in Neuroengineering, Edmond and Lily Safra International Institute of Neuroscience, Santos Dumont Institute, Macaiba, Brazil; 3 PUCRS, Escola de Ciências, Laboratório de Biotecnologia Vegetal, Porto Alegre, Brazil

**Keywords:** hepatic fibrosis, HSCs, Baccharis, PPAR-gamma, phenolic compounds

## Abstract

The therapeutic potential of *Baccharis anomala *DC. extracts was evaluated through its cytotoxic and antiproliferative effect and their phenotypic reversion property in activated hepatic stellate cells (HSCs). *Baccharis anomala* is distributed in Brazil (southeastern and south regions) and used for diuretic effect in folk medicine. Four fractions were obtained from the fractionation of the methanolic extract. Fractions III and IV decreased cell proliferation without increasing cell necrosis markers levels and induced cell cycle arrest in G1 phase. Fraction III induced phenotypic reversion through PPAR-γ activation pathway, while fraction IV did not alter PPAR-α/γ expression levels, suggesting that there is an independent PPAR-α/γ pathway involved. Hydroxybenzoic, chlorogenic and coumaric acids were identified. Fractions III and IV showed antiproliferative effect and ability to induce reversion of activated phenotype of HSCs.

## Introduction

Hepatic fibrosis is associated with chronic liver damage and can be caused by different factors such as chronic viral hepatitis, alcohol abuse, toxins, metal accumulation and hereditary diseases (Iredale, 2008[22]). In response to liver injuries, the hepatic stellate cells (HSC) gradually transdifferentiate to an activated phenotype (aHSC) and consequently liver fibrosis. aHSC lose the ability to store vitamin A, increase expression of a-smooth muscle actin (α-SMA) and profibrotic genes, and acquired a larger and polygonal shape. In addition, aHSC synthesize collagen, a component of the extracellular matrix, and produce contractile movements after stimulation, as occurs in wound healing. These characteristics may lead to the destruction of hepatic tissue architecture and, in severe cases, to the development of cirrhosis and liver failure (Friedman, 2008[13]; Gressner, 1998[15]; Wynn and Ramalingam, 2012[42]). Therefore, it is widely accepted that activation of HSC is a key factor in the pathogenesis of liver fibrosis. 

Under normal conditions, the quiescent phenotype of the HSC is maintained by the transcription factors peroxisome proliferator-activated receptors (PPAR) such as PPAR-α, PPAR-β/δ and PPAR-γ. The PPAR-γ transcription factor has been considered as one of the most important factors for the regulation of adipogenesis in HSCs (Guimaraes et al., 2007[16]; Tsukamoto et al., 2006[38]) and maintenance of quiescent phenotype (Moraes et al., 2014[28]; Mesquita et al., 2013[27]). Moreover, studies also revealed that modulation of lipid metabolism could occur via PPAR-α (Chen et al., 2015[8]). Therefore, the inhibition of HSC activation seems to be an effective strategy for therapy of liver fibrosis. 

GRX cell line is an immortalized lineage of liver cells obtained from hepatic granulomas of mice infected with *Schistosoma mansoni *and it is considered an excellent model for representation of liver fibrosis *in vitro *(Borojevic et al., 1985[4])*. *GRX lineage normally presents the myofibroblast transitional phenotype, being between the quiescent and activated stages (Herrmann et al., 2007[20]). In order to activate myofibroblasts the presence of profibrogenic cytokines and oxidative stress are necessary that cause cytoskeleton reorganization as well as an increase in collagen production. The quiescent phenotype expression can be induced when the transitional myofibroblast is exposed either to retinol, indomethacin or β-carotene (Guimaraes et al., 2006[17]).

The N-acetylcysteine, a drug mainly known for its antioxidant effect, can be highlighted amongst the most used drugs for controlling fibrosis, and it is often used in cases of paracetamol intoxication (Vargha et al., 2014[39]; Heard, 2008[18]; Lauterburg et al., 1983[25]). Plants have been pointed out as natural resources for traditional medicine and for the modern pharmaceutical industry. Besides, the use of plants able to inhibit the proliferation of activated HSCs has a great potential in reversing fibrosis. The beneficial effects are due to compounds produced by the plant, known as secondary metabolites, and their production and accumulation by the plant are related to the interaction of the plant with the environment. Factors such as temperature, predation and luminosity can substantially influence the phytochemical profile (Sartor et al., 2013[35]). Phenolic compounds represent one of the main studied classes of secondary metabolites, mainly due to their antioxidant activity (Balasundram et al., 2006[3]; Neuhouser, 2004[29]). The genus *Baccharis *has approximately 500 known species (Abad and Bermejo, 2007[1]; Budel et al., 2005[5]) and many species are used in folk medicine in form of teas, alcoholic beverages or macerated, for the treatment of various diseases, such as gastrointestinal diseases, liver diseases, inflammation, diarrhea, fever, infections and diabetes (Campos et al., 2016[6]; Hocayen et al., 2016[21]). Among the known species of the genus, about 90 % can be found in South America, being more common in Brazil, Argentina, Uruguay, Chile, Colombia and Mexico. The main constituents found in the *Baccharis *genus are phenolic compounds and terpenoids (Campos et al., 2016[6]). The species *B. megapotamica, B. incarum, B. trimera, B. trinervis, B. salicifolia, B. uncinella, B. coridifolia, B. dracunculifolia, B. grisebachii *and *B. tricuneata *have been extensively studied for their chemical composition (Verdi, 2005[41]).

*Baccharis anomala *DC., also known as “cambará-de-cipó” or “parreirinha”, is geographically distributed in the southeastern (Minas Gerais, São Paulo) and south (Paraná, Santa Catarina, Rio Grande do Sul) regions of Brazil (Heiden and Schneider, 2010[19]), although it is also found in Argentina, Uruguay, and Paraguay. The aerial parts of *B. anomala *are mainly used for its diuretic effect in folk medicine (Budel et al., 2005[5]; Alice et al., 1985[2]; Garlet, 2000[14]). Since many species of the Baccharis genus have beneficial effects on liver diseases and also in inflammatory processes, the identification of the therapeutic activity of other species of this genus can be promising. As few studies on the phytochemical composition and medicinal properties of this species are currently available, the aim of this study was to evaluate the cytotoxicity, antiproliferative and deactivation effects of the methanolic extract and fractions of *B. anomala *on activated hepatic stellate cell line GRX. 

## Material and Methods

### Plant material and extraction

Leaves from *Baccharis anomala *DC. were collected in São Francisco de Paula in the state of Rio Grande do Sul (Southern Brazil; latitude, 29◦29S; longitude, 50◦11 W; 950 m). The specimen was deposited in the Herbarium of the Science and Technology Museum of the Pontifícia Universidade Católica do Rio Grande do Sul (Herbarium MPUC) under the voucher specimen number Herbarium 3354. Plant material was dried, powdered and then stored at -20 ºC until use. Extracts were prepared by combining 3 g of dried plant material with either 100 mL of distilled water (aqueous extract), 100 mL of ethanol 96 % (ethanolic extract) or 100 mL 80 % aqueous methanolic solution (80:20; methanolic extract). Aqueous and ethanolic extracts were agitated for 5 and 72 h at room temperature, respectively. Methanolic extract was kept in ultrasonic bath for 15 min at room temperature. Extracts were then centrifuged for 10 min at 4000 g, the supernatant was collected and dried in a rotary evaporator. For the experiments with extracts and fractions, the dry material resulting from evaporation of the respective solvents in the rotary evaporator was resuspended in culture Dulbecco's Modified Eagle's Medium (DMEM). The negative control consisted of culture medium and GRX cells with no extract added. 

### HSC culture 

Immortalized hepatic stellate cell GRX was obtained from the Rio de Janeiro Cell Bank and presents a myofibroblast transitional phenotype. Cells were maintained in culture medium (DMEM) supplemented with 5 % fetal bovine serum (FBS) (GIBCO), 1 % penicillin and streptomycin (Invitrogen) and pH 7.4. HSC were incubated with *Baccharis* *anomala* extracts for 72 hours at 37 °C in a humidified atmosphere of 5 % CO_2_. Results using GRX derived from at least three replicates per experimental condition.

### Antiproliferative and cytotoxicity evaluation of aqueous, ethanolic and methanolic extracts

Proliferation and cell viability was assessed determining living cell numbers by cell count with Trypan blue exclusion method. GRX cells were seeded in a 24-well tissue culture plate (3x10^3^ cells/well) and incubated with *B. anomala* crude extracts at concentrations of 25, 50, 500 µg/mL. N-acetylcysteine (NAC; 400 µg/mL), a well-known medicine used in the treatment of hepatic fibrosis, was used as a positive control. For all experiments, the negative control consisted of GRX cells on culture medium. Methanolic extract was chosen to conduct the experiments, due to the most significant antiproliferative effect. 

### Fractionation of methanolic extract by chromatography column

Methanolic extract was fractionated by chromatography on a silica column, using silica gel 60 (Merk) as stationary phase and the mobile phase consisted in eluents of increasing polarity (v/v): dichloromethane (100:0), dichloromethane: methanol (95:5), dichloromethane: methanol (90:10), methanol (100:0) and methanol: water (80:20). Four different fractions were obtained, therein named FI, FII, FIII and FIV. Fractions were filtered, dried and weighed for their use in the experiments. 

### Evaluation of antioxidant activity of the fractions from methanolic extract 

Fractions from the methanolic extract were analyzed for its antioxidant activity. The reduction of DPPH (2,2-diphenyl-1-picrylhydrazyl) through electron transfer by the action of an antioxidant was measured by spectrophotometry in an ELISA reader at the wavelength of 515 nm. All samples analyzed were dissolved in methanol 100 %. Ascorbic acid (550 μg/mL) was used as a positive control for antioxidant activity.

### Antiproliferative effect and cytotoxicity of FIII and FIV on GRX cells 

In order to evaluate the cytotoxity of fractions and their effect on cell proliferation of GRX cells, cells were seeded in a 24-wells tissue culture plate (3x10^3^ cells/well) and incubated with FIII and FIV at concentrations of 1.25, 2.5, 5, 50, and 100 µg/mL. NAC (400 µg/mL) was used as positive control. Proliferation was assessed by cell count with Trypan blue exclusion method. The evaluation of cytotoxicity was performed using a lactate dehydrogenase (LDH) kit (LabTest, Brazil) in the culture supernatants. As LDH is a known enzyme related to membrane cell damage, its activity was measured by a colorimetric assay at 420 nm and compared with the negative control. For the control of total cell lysis, 5 % Tween was used. Analyses were performed after 72 h of treatment with fractions.

### Cell cycle evaluation

Cell cycle arrest was evaluated using 7-AAD staining. GRX cells were seeded into 24-well plates at 3x10^3^ cells/well and treated either with FIII at concentration of 50 µg/mL, FIV at 5 µg/mL or NAC at 400 μg/mL. Cells were collected by trypsinization, washed twice with PBS and then, while vortexing, 2.5 mL of ethanol 70 % was added per sample. Cells were incubated at -20 ºC for 2 h and then washed twice with PBS solution to remove ethanol. Cells were centrifuged and resuspended in 100 µL of stain buffer and 4 µL of 7-AAD, and incubated for 15 min at room temperature. Samples were analyzed by flow cytometry to identify the cell cycle phases. Data were analyzed using FlowJo 7.6.5 software (Tree Star). Analysis allows discrimination among Sub-G1, Go/G1, S, G2/M.

### Detection of lipid droplets in aHSC

Phenotypic reversion in hepatic stellate cells was observed using Oil Red assay. Cells were plated in a 24-well tissue culture plate (3x10^3^ cells/well), and 72 h after treatment with FIII and FIV, cells were fixed with 10 % formaldehyde for 1 h and stained with Oil Red-O (ORO; Sigma Chemical). Intracellular lipid accumulation was observed after 30 min, using an inverted light microscope at magnification of 400x. For estimation of lipid accumulation, the ORO within the lipid droplets was extracted using isopropanol and the absorbance was read at 492 nm using ELISA plate reader. Specific lipid content was calculated as the ratio of absorbance value obtained for ORO and number of cell count.

### Morphological analysis of aHSC nucleus for apoptosis detection

The nuclear morphology of cells was evaluated using the cell-permeable DNA dye 6-diamidino-2-phenylindole (DAPI). Cells were seeded at a cell density of 3x10^3^ cells/well in 24-well plates and incubated for 24 h at 37 ºC in a 5 % CO_2 _incubator. Cells were then treated with FIII and FIV fractions, NAC at 400 μg/mL and Cisplatin at 2.5 µM (positive control of apoptosis). After 72 h incubation, cells were fixed with 4 % paraformaldehyde for 2 h. Fixed cells were permeabilized with 0.3 % Triton X-100 in phosphate buffered saline (PBS) for 30 min and subsequently stained with a solution containing 300 nM of DAPI for 2 min. Incubation was carried out at room temperature and cells were photographed using a fluorescence microscope (IX71, Olympus).

### RNA extraction and real time quantitative PCR

Total RNA was extracted from cells using TRIzol reagent (Invitrogen). RNA was reversely transcribed into cDNA, using Superscript III First-Strand Synthesis SuperMix (Invitrogen) according to the manufacturer's instructions. Relative expression levels of α-SMA, PPAR-α and PPAR-γ with respect to glyceraldehyde 3-phosphate dehydrogenase (GAPDH) was quantified by qRT-PCR, conducted on Step One Applied Biosystems. The reaction was catalyzed by SYBR Green I (Applied Biosystems- Thermo Fisher Scientific) kit according to the manufacturer´s instructions.

### Assessment of aHSC cell contraction by collagen gel analysis

Collagen from rat tail tendon was extracted and prepared as described by Rajan et al. (2006[34]). Collagen gels (125 μl of 4x DMEM and 125 μl of 4 mg/mL Rat Tail Tendon Collagen) were impregnated with 1x10^5^ cells resuspended in 250 μl of PBS. Gels were added to a 24-well plate, left to polymerize for 30 min at 37 °C, detached and suspended in 600 μL of DMEM with 5 % fetal bovine serum (FBS) alone or with FIII, FIV and NAC. Images were made after 24 h and the surface of the area of each gel was determined as percentage of well area, using ImageJ (http://rsb.info.nih.gov/ij). 

### Determination of phenolic compounds

Phenolic compounds were analyzed in FIII and FIV by HPLC. Chromatographic analysis was performed using a Sykam System S600 equipped with a MetaSil ODS reverse phase column (5 μm, 150 x 4.6 mm) and DAD UV-VIS detector set at 280 nm). The column was maintained at 40 °C, and the injected sample volume was 20 μL. Chromatographic data were obtained and processed by the Clarity Chromatography Software® system. The following eluents were used: 2 % phosphoric acid in water (eluent A) and methanol (eluent B). The gradient elution program was 10 % eluent B from 0 to 10 min, 20 % to 80 % B from 10 to 25 min, 80 % to 100 % B from 25 min to 32 min and 100 % B from 32 to 35 min. The flow rate was maintained at 1 mL/min. Compounds were identified based on the retention time of pure standard and quantified by reference to peak areas of the standard curves.

### Statistical analysis

Data are reported as mean ± SD. Data were analyzed by one-way analysis of variance (ANOVA) followed by Tukey multiple comparison test at a significance level of *p* < 0.05. The statistical program used was GraphPad Prism Version 5.00.

## Results

### Effect of aqueous, ethanolic and methanolic crude extracts of B. anomala on cell number

**Figure 1:**
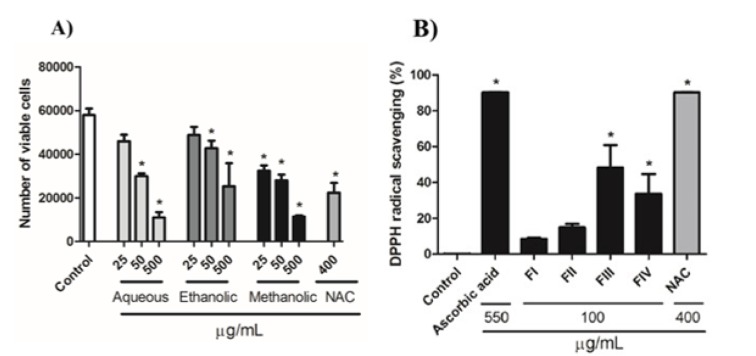
A) Effect of crude extracts of *Baccharis anomala* on cellular proliferation of GRX cells during 72 h of treatment. Cellular proliferation and cell viability was assessed determining the number of viable cells by Trypan blue exclusion method. Data represent the mean ± SD (n = 3). Results were expressed as cell number. * P<0.05 compared with negative control. B) Antioxidant activity of the fractions obtained from the methanolic extract at 100 µg/mL. The antioxidant activity of NAC was evaluated at 400 μg/mL. Ascorbic acid was used as the control for antioxidant activity at 550 µg/mL. The results are presented as percentage of DPPH reduction in relation to the control group. Data represent the mean ± SD (n = 3). * P < 0.05 compared with negative control

Results showed that all extracts reduced cell proliferation within 72 h of treatment at 50 and 500 µg/mL. However, only the methanolic extract decreased the cell number at 25 µg/mL when compared with control group (Figure 1A(Fig. 1)). As the methanolic extract presented the most significant result, it was selected for the next experiments. 

### Identification of antioxidant activity by DPPH in fractions obtained by column chromatography

The antioxidant potential of each fraction obtained in chromatography column was determined with the DPPH assay. At concentration of 100 µg/mL, fractions FIII and FIV showed significant antioxidant activity when compared to control group, while no significant activity was observed with FI and FII. NAC exhibited a great antioxidant activity reaching the same level of activity as ascorbic acid (Figure 1B(Fig. 1)). 

### FIII and FIV decrease cell proliferation without cytotoxicity

**Figure 2:**
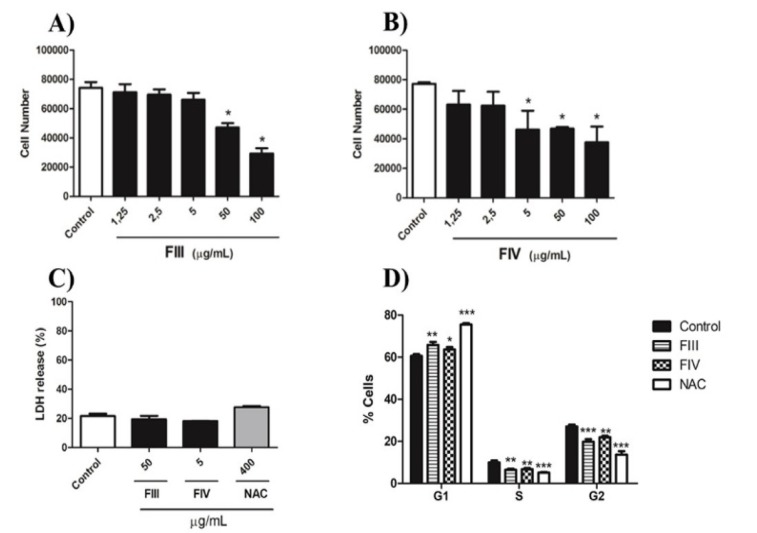
Effect of fractions on GRX cells proliferation during 72 h of treatment. (A) FIII; (B) FIV at concentrations of 1.25, 2.5, 5, 50, and 100 µg/mL. Cellular proliferation assessed by Trypan blue exclusion method. Data represent the mean ± SD (n = 3). Results were expressed as cell number.* P < 0.05 compared with control. (C) Cytotoxicity of fractions III and IV was evaluated by measuring LDH release levels in the supernatant after 72 h of treatment. Data represent the mean ± SD (n = 3). The results were presented as percentage of LDH release in the supernatant in relation to the total content of LDH culture obtained by cell lysis. (D) Cell cycle arrest in GRX cells were evaluated by 7-AAD. FIII at 50 µg/mL, FIV at 5 µg/mL and NAC at 400 µg/mL. Samples were analyzed by flow cytometry to identify cell cycle phases. Results were presented as percentage of cells at each phase of the cell cycle. Data were analyzed using FlowJo 7.6.5 software (n=3). ***P < 0.0001, **P < 0.001 and * P < 0.05 compared with negative control.

Fractions FIII and FIV were selected for evaluating their effect on GRX cell proliferation based on their high antioxidant activity. FIII significantly decreased cell number compared to negative control at concentration of 50 and 100 µg/Ml (Figure 2A(Fig. 2)). Moreover, treatment with FIV resulted in reduction of proliferation when 5, 50 and 100 µg/mL were used (Figure 2B(Fig. 2)), evidencing a stronger effect of FIV on GRX cell line when compared to FIII. Thus, based on the minimum concentration for a significant reduction of cell number, fractions were used at 50 µg/mL and 5 µg/mL for FIII and FIV, respectively. The supernatant of cell culture was used to evaluate the cytotoxicity of the fractions FIII and FIV through LDH assay 72 h after treatment. Results showed that the fractions were capable to reduce cell number without causing cell damage, indicating that both fractions show no cytotoxicity on GRX cells at the concentrations tested (Figure 2C(Fig. 2)). In an attempt to understand the decrease in cell number after treatment with FIII and FIV, evaluation of the cell cycle was carried out. Both fractions and NAC significantly induced GRX cell cycle arrest in G1-phase when compared to the negative control. Indeed, FIII promoted a significant increase in cycle arrest when compared to FIV (Figure 2D(Fig. 2)). 

### Visualization of apoptotic cells by DAPI

**Figure 3:**
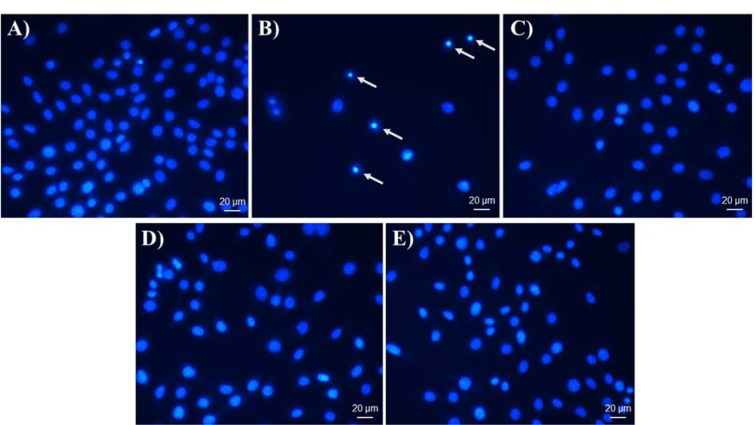
DAPI nuclei staining. Effect of FIII and FIV, NAC and Cisplatin on apoptosis of GRX cells analyzed by nuclear morphology. (A) Negative control group, (B) Cisplatin at 2.5 µM, (C) NAC at 400 µg/mL, (D) FIII at 50 µg/mL and (E) FIV at 5 µg/mL. Cells were treated for 72 h. Cisplatin was used as positive control of apoptotic inducer; apoptotic cells demonstrated nuclear condensation (arrows).

In order to investigate whether the decrease in cell proliferation caused by FIII and FIV was due to apoptosis, the nuclei were stained with DAPI. The morphological profile indicated nuclear condensation and fragmentation suggesting a pro-apoptotic effect of Cisplatin on GRX cells (Figure 3B(Fig. 3)). However, cells treated with NAC as well with FIII and FIV showed no alteration on morphological profile (Figure 2,C,D,E(Fig. 2)).

### FIII and FIV induce phenotypic reversion on aHSC 

**Figure 4:**
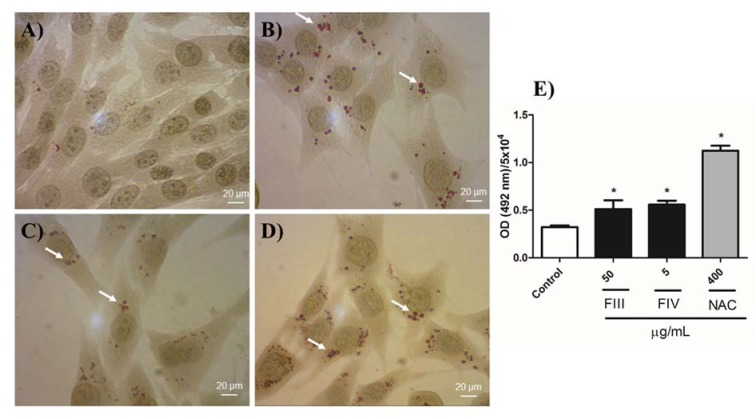
Oil Red-O (ORO) staining and lipid quantification of GRX cells at 72 h. (A) Negative control, (B) FIII at 50 µg/mL, (C) FIV at 5 µg/mL and (D) NAC at 400 µg/mL, 400x magnification. Lipid droplets indicated by arrows. (E) Lipid quantification. Results are shown as the absorbance value obtained for ORO adjusted for number of 5x10^4^ cells. Results are expressed as mean ± SD (n=3). * P < 0.05 compared with negative control

The ability of fractions to revert GRX cell phenotype by inducing the accumulation of lipids in the cytoplasm was investigated. Compared to the negative control, GRX cells treated with the fractions showed increased ability of storing fat in the cytoplasm (Figure 4B, C(Fig. 4)). The NAC treatment also induced the accumulation of lipids in the cytoplasm (Figure 4D(Fig. 4)). Lipid accumulation was quantified by absorbance at 492 nm and confirmed the positive result of the fractions on storing lipids by GRX cells (Figure 4E(Fig. 4)). Furthermore, GRX cells treated with FIII, FIV and NAC lost their elongated and parallel strand appearance and acquired a larger and polygonal shape, whereas control cells maintained their myofibroblast-like morphology. 

### Expression of α-SMA, PPAR-α and PPAR-γ

**Figure 5:**
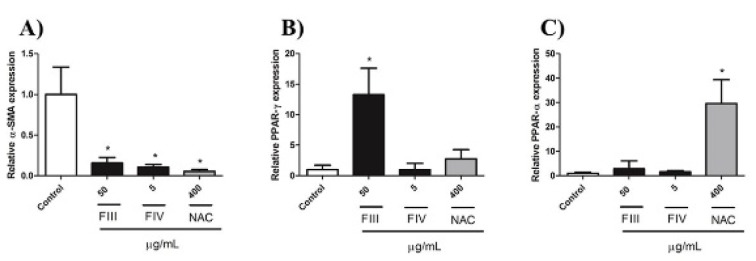
Effect of FIII (5 µg/mL), FIV (50 µg/mL) and NAC (400 µg/mL) on (A) α-SMA, (B) PPAR-ɣ and (C) PPAR-α expression of GRX cells treated for 72 h. Results are presented as relative expression of GAPDH. Data are expressed as mean ± SD (*n*=3). *P < 0.05 vs. negative control

The relative expression of α-SMA decreased in all treatments in relation to negative control group (Figure 5A(Fig. 5)). As ORO staining revealed the formation of fat droplets, the differential expression of PPAR-α and PPAR-γ was assessed in order to elucidate the pathway of the phenotypic reversion mechanism (Figure 5B, C(Fig. 5)). Although GRX cells treated with NAC showed a significant increase in PPAR-α expression, treatment with FIII and FIV did not show significant result. No changes were observed in PPAR-γ expression in treated groups with FIV and NAC, only treatment with FIII increased the relative expression of PPAR-γ when compared to negative control (Figure 5B(Fig. 5)), suggesting that another pathway might be involved in accumulation of lipids droplets on cytoplasm of GRX cells treated with FIV.

### Collagen gel contraction assay

**Figure 6:**
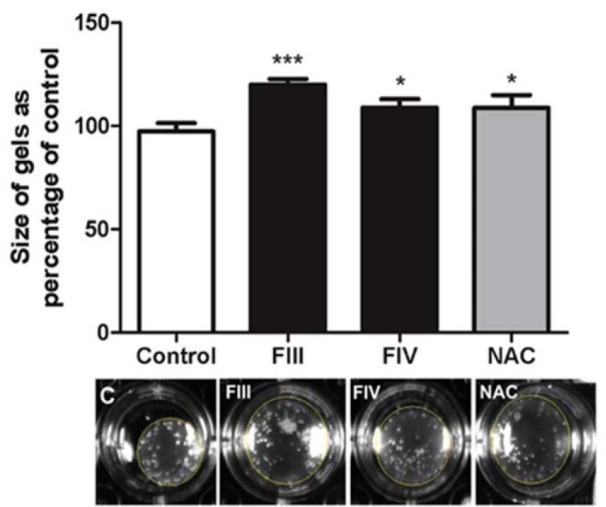
Cell contraction assessed by collagen gel assay in GRX cells. Mean ± SD are shown. n= 4 per group. ***P < 0.001 vs.*P < 0.05 vs. negative control, by one-way ANOVA and Tukey test

After 24 h of treatment, it was possible to observe that the area occupied by the collagen gel that received treatment with the fractions was larger in relation to the area of the control group. Therefore, it indicates that both fractions (FIII and FIV) were able to decrease cell contraction, whereas a greater contraction was detected in the control group cells likely due to its activated phenotype (Figure 6(Fig. 6)).

### Determination of phenolic compounds

Identification and quantification of selected phenolic compounds in FIII and FIV were performed by HPLC analysis. In FIII, chlorogenic acid (26.66 %), coumaric acid (1.39 %) and hydroxybenzoic acid (0.78 %) were the identified phenolic compounds. In FIV, only chlorogenicacid (1.9 %) and coumaric acid (0.53 %) were detected. The retention times and percentages of each compound identified in FIII and FIV are shown in Table 1(Tab. 1).

## Discussion

The major finding of the current study is that *Baccharis anomala *DC. promotes deactivating effect in the phenotype of activated HSC evidenced by the reduction of proliferation, induction of lipid drops accumulation and decrease in α-SMA expression.

Studies have shown that the consumption of foods rich in phenolic compounds can prevent disease development (Neuhouser, 2004[29]). It has been also demonstrated the health benefits and the applicability of these compounds to the development of new therapies, indicating that the search for natural sources of phenolic compounds is a promising alternative. Some groups of plants have gained visibility due to the high production and accumulation of phenolics, such as the genus *Baccharis *(Budel et al., 2005[5]; Campos et al., 2016[6]). This genus comprises many species used in the popular medicine by several cultures of South America. Out of the 500 known species approximately 30 % have the phytochemical composition determined (Abad and Bermejo, 2007[1]). Thereby, the search for species with potential for the development of new applicability and therapies is worthwhile. 

The present study investigated the cytotoxicity and antiproliferative effect of leaf extract from *Baccharis anomala *DC. on hepatic stellate cells, as well as the ability of revert the phenotype of such cell. In addition, selected phenolic compounds were identified in the extract and their possible relation to the biological effects of the extract is suggested. 

Amongst the *B. anomala *extracts tested, the methanolic showed a significant reduction of cell proliferation in the lowest concentration tested (25 µg/mL). This result led us to look for the main compounds present in the methanolic extract. To this end, the extract was fractionated by column chromatography and four fractions were obtained. Methanolic fractions (FIII and FIV) showed high antioxidant activity, a chemical characteristic that seems to be promising for treatment of hepatic fibrosis (Francia et al., 2016[12])

FIV fraction was able to significantly decrease cell proliferation at 5 µg/mL, while FIII was efficient at 50 µg/mL. These doses were selected for cytotoxicity assessment by LDH test, because the activity of LDH in cell culture supernatant is a marker of cellular injury. Cytotoxicity was not observed from FIII, FIV and NAC, showing that the decrease in proliferation was not consequence of necrosis. 

A study with *Baccharis articulata* revealed the ability of aqueous extract to induce apoptosis in human peripheral blood mononuclear cells (Cariddi et al., 2012[7]). Moreover, a study conducted with the polyphenol resveratrol on activated hepatic stellate cells demonstrated the ability to induce apoptosis through the evaluation performed with DAPI staining (Souza et al., 2008[37]). To verify a possible pro-apoptotic mechanism action of the methanolic fractions that could lead to a decrease in proliferation of GRX cells, nuclear morphology was visualized through the DAPI staining. As positive control of apoptosis cisplatin was used, which is a well-known chemotherapeutic drug used for treatment of numerous human cancers (Dasari and Tchounwou, 2014[9]). Cisplatin form adducts on DNA, causing damage and subsequently, inducing apoptosis (Siddik, 2003[36]). The results showed that the cells treated with FIII, FIV and NAC showed no alteration in their nuclear morphology in relation to the control group. On the other hand, in addition to significantly reducing cell proliferation, cisplatin has shown nuclei with a condensed morphology, indicating the formation of apoptotic bodies. This led us to believe that the decrease in cell proliferation was not due to death by apoptosis, driven our investigation to the action of FIII and FIV methanolic fractions on the cell cycle.

Several studies have reported the action of phenolic compounds on the cell cycle (Jafari et al., 2014[23]). Cell cycle is modulated and controlled by complexes of cyclins and cyclin-dependent kinase enzymes (CDKs). The expression of these enzymes can be regulated by simple phenols or complex polymeric structures, such as tannins (Jafari et al., 2014[23]). Treatment of HL-60 cells with gallic acid resulted in G0/G1 phase arrest, which was associated with up-regulation of p21 and p27 (Yeh et al., 2011[43]). Thus, the investigation of the cell cycle seems to be necessary to understand the activity of these compounds on cell proliferation. Our results showed that both fractions and NAC induces cell cycle arrest in G1-phase in GRX cells. Cell cycle arrest in G1-phase observed in NAC treatment is in agreement with the literature, since NAC mediates cell cycle arrest through a Sp1-dependent mechanism (Kim et al., 2001[24]). The fundamental point of this evaluation is the stop of the cycle in phase G1, since all the proliferation is suffering a delay. It is noteworthy that in G1 there was a significant increase in the number of cells in relation to the control (p <0.001 and 0.05). We believe that it represents a significant decrease in cell proliferation, although it is a small difference. 

Quiescent state HSCs accumulate retinol (vitamin A) in their cytoplasm, therefore they are also known as lipocytes, functioning in the maintenance of hepatic tissue, through synthesis of proteins responsible for formation and degradation of ECM components (Iredale, 2008[22]; Borojevic et al., 1985[4]). This study showed lipid deposits in intracellular content of GRX cells as well as changes in cell morphology observed in phase contrast microscopy. The GRX cells treated with FIII, FIV and NAC lost their elongated and parallel strand appearance and acquired a larger and polygonal shape, while control cells preserved their myofibroblast-like morphology, devoid of large lipid droplets. Quantification of the total lipid content was possible through the absorbance corrected by number of 5x10^4 ^cells, which confirmed the significant increase in lipid production in cells treated with the fractions and NAC during 72 h. Our results show that FIII and FIV obtained from the methanolic extract showed antiproliferative effect and the ability to deactivate HSCs, transforming the fibroblastic phenotype into quiescent cells. 

There are three peroxisome proliferator-activated receptors (PPARs) members of the nuclear hormone receptor family of ligand-activated transcription factors and consisting of three different isoforms: PPAR-α, PPAR-β/δ and PPAR-γ, involved in the regulation of lipid synthesis. PPAR-γ is a transcription factor that is related to induction of quiescent phenotype in HSCs (Guimaraes et al., 2007[16]; Tsukamoto et al., 2006[38]). Currently, there are studies on molecules capable of inducing the expression of PPAR-α, leading to deactivation of HSCs through pathways independent of PPAR-γ expression (Chen et al., 2015[8]). To investigate the mechanism involved in restoring the quiescent phenotype, the PPAR-α and PPAR-γ expression was evaluated by qPCR. The results showed that expression of PPAR-γ was increased with FIII treatment but did not alter by treatment with FIV and NAC. The non-alteration of PPAR-γ expression in NAC treatment appears to be in agreement with the literature and the obtained results, since its effect is mainly related to the expression of PPAR-α (Paintlia et al., 2008[32]). The increase in PPAR-α expression was only statistically significant in the treatment with NAC. As treatment with FIII increased the expression of PPAR-γ we believe that might be the mechanism by which induction of quiescent phenotype occurs in HSCs. Thus, it is possible that the restoration of the quiescent phenotype of the HSCs treated with the FIV could be occurring by a PPAR- α/γ-independent pathway. 

Analysis by HPLC performed in FIII allowed the identification of the major phenolic compounds, using internal phenolic standards. It was possible to identify in FIII the presence of chlorogenic, hydroxybezoic and coumaric acids. The chlorogenic acid was the most abundant phenolic in FIII. We cannot ignore the possibility of presence of others compounds, however they were not identified by the used method. Some of the phenolic compounds identified by HPLC analysis are in agreement with the phytochemical composition of species found in the genus (Campos et al., 2016[6]). Species that have several studies regarding their chemical composition, such as, *B. dracucunlifolia*, are known to produce a large amount of phenolic compounds, such as caffeic acid, cinnamic acid, drupanin, baccharin, artepillin C and p-coumaric acid (Campos et al., 2016[6]; Hocayen et al., 2016[21]). Studies have demonstrated the antiviral, antibiotic, antidiabetic, antimicrobial, hepatoprotective properties and antiproliferative activity, *in vitro* and *in vivo* models, using crude or fractionated extracts of this species (Campos et al., 2016[6]; Hocayen et al., 2016[21]; Paintlia et al., 2008[32]; Pereira et al., 2016[33]). Similarly, aqueous and ethanolic extracts of *B. trimera* showed antiulcerogenic activity, anti-inflammatory and anthelmintic activity (Oliveira et al., 2014[31]; Dos Reis Liveiro et al., 2016[11]; Menezes et al., 2016[26]; Nogueira et al., 2011[30]) due to the presence of phenolic compounds such as chlorogenic acid, rutin, ellagic acid, rosmarinic acid, luteolin and quercetin (Menezes et al., 2016[26]). *B. uncinella* is often used as anti-inflammatory in folk medicine (Zalewski et al., 2011[44]). Studies on the chemical composition of this species have revealed the presence of hispidulin, caffeic acid, chlorogenic acid and pectolinarigenin (Campos et al., 2016[6]). Moreover, benzoic acid and it derivatives, such as p-hydroxybenzoic acid, found in both fractions, are phenolic compounds that exhibit a well-known antioxidant activity and are widely used as food preservatives (Ding et al., 2015[10]). Chlorogenic acid belongs to the group of hydroxycinnamates, being the most abundant element in human diet from this group. Chlorogenic acid is the main phenolic compound in coffee and it consists of the conjugate ester of caffeic, ferulic or p-coumaric acids with quinic acid. The molecule shows a strong antioxidant activity (Zhao and Moghadasian, 2010[45]) and it is the third most abundant compound in FIII. Thus, we believe that at least in part, the antioxidant, antiproliferative and the reversion of phenotype effects observed in the treatments with the methanolic fractions are due to the presence of hydrobenzoic acid and chlorogenic acid. In the FIV, chlorogenic and coumaric acids were detected in smaller proportions than in the FIII fraction. For this reason, we believe that there is a molecule or synergism of molecules which could be responsible for the strong antiproliferative effect observed with FIV. Further investigations are necessary to identify these molecules. 

During the development of liver fibrosis occurs the process of transformation HSCs quiescent (lipocytes) into myofibroblasts and this process can result in alterations in contraction capacity of cells and, therefore, lead to a physiological effect in the liver, an effect that can result in a more serious problem, such as portal hypertension (Iredale, 2008[22]). The deactivation of the HSCs cells is an important therapeutic target. With the deactivation of the HSCs, the production of components of extracellular matrix decreases, resulting in reduction of contraction. In our work, this reduction was observed in collagen gel with cells treated with FIII and FIV of methanolic extracts of *B. anomala.*

Notwithstanding FIII and FIV presented some components in common, they vary greatly in quantity. FIV was able to decrease proliferation and reverse cell phenotype with a concentration ten-fold lower than FIII. However, FIII presented better results in cell cycle arrest and antioxidant activity, showing that both fractions have potential for the treatment of liver fibrosis, even though they own distinctive features. FIV appears to be more purified than FIII, since it was the last fraction to be collected in column chromatography. Thereby, we believe that the predominance of a certain compound in this fraction may be responsible for the effect observed in the HSCs in a lower concentration. 

Further studies will be necessary to compare the effect of the fractions with the isolated molecules, in order to better understand the effect that each one exerts on HSCs and how they could act concomitantly and thus search for mechanisms of action. Phenolic acids are natural compounds that can act synergistically with other molecules present in the plant extract. Therefore, their effects can complement each other acting more effective on a biological system and likely do not exhibit as significant biological activity when used isolated as they do in synergism (Vaz et al., 2012[40]). This study was able to evaluate the cytotoxicity, phenotypic reversion and antiproliferative effect of the fractions obtained from methanolic extract of *Baccharis anomala* and showed the potential for the treatment of liver fibrosis.

## Acknowledgements

Authors are thankful to the Institute of Toxicology and Pharmacology (INTOX) for providing help and technical support. This study was financed in part by the Coordenação de Aperfeiçoamento de Pessoal de Nível Superior - Brasil (CAPES) - Finance Code 001. License for research on Brazil's biodiversity, CNPq # 010852/2014-0.

## Conflict of interest

We confirm that there are no known conflicts of interest associated with this publication and there has been no significant financial support for this work that could have influenced its outcome.

## Figures and Tables

**Table 1:**
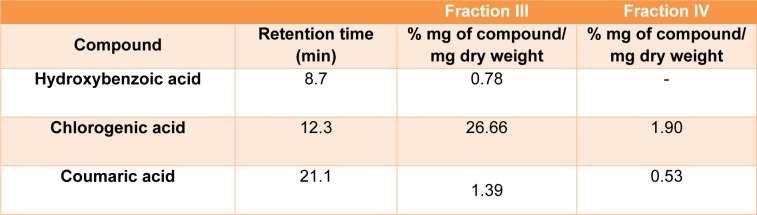
HPLC analysis of phenolic compounds in *B. anomala *obtained fractions. Results expressed as mean ± standard deviations (SD) of three samples
